# Cardiovascular Risk Factors Among Healthcare Providers at the Bamenda Regional Hospital, Bamenda, Cameroon

**DOI:** 10.7759/cureus.79709

**Published:** 2025-02-26

**Authors:** Etienne Ngeh Ngeh, Ayuba Berinyuy Wiysahnyuy, Emmanuel Tito

**Affiliations:** 1 Physiotherapy, Sheffield Hallam University, Sheffield, GBR; 2 Physical Therapy, St. Louis University Institute of Health and Biomedical Sciences, Douala, CMR; 3 Medicine, Johns Hopkins University School of Medicine, Baltimore, USA

**Keywords:** cardiac risk factors and prevention, cardio vascular disease risk reduction, healthcare profession, health improvement, health improvement card, public health education

## Abstract

Objective

This study aimed to assess cardiovascular risk factors (CVRFs) among healthcare professionals (HCPs) practicing at the Bamenda Regional Hospital (BRH) in Bamenda, Cameroon.

Methodology

This was a hospital-based cross-sectional study involving HCPs practicing at the BRH. Data on lifestyle risk factors were collected across several units and services of the BRH by using a modified Health Improvement Card (HIC).

Results

A total of 237 participants were included in the final analysis; most were female (59.1%). The mean age of the sample was 30.1 ± 5.8 years. Based on the HIC, most HCPs were in the medium-risk zone (54.0%) for diet; in the high-risk zone for physical activity (68.4%); and in the low-risk zone for tobacco use and alcohol consumption (97.9% and 82.3%, respectively). The risk of developing cardiovascular diseases (CVDs) increases from a low to high-risk level. The mean HIC score was 10.3 ± 1.8 in males), and 10.2 ± 1.7 in females (p=0.781). The age group of 20-30 years had the lowest HIC score, with a mean of 9.9 ± 1.6; those over 40 years had higher HIC scores, with a mean of 11.5 ± 1.4 (p=0.000). When HIC CVRFs were quantified (higher score indicates higher risk), the HIC scores were highest in physicians (11.0 ± 1.8), followed by pharmacists (11.0 ± 1.4) and nurses (10.5 ± 1.7), and lowest in physiotherapists (8.0 ± 1.1) (p=0.000). Regarding socioeconomic status, the mean HIC scores were highest for the upper class (11.5 ± 1.3), followed by the middle (10.0 ± 1.9) and lower classes (10.0 ± 1.7) (p=0.013).

Conclusions

Most participants were in the low-risk zone for BMI and alcohol and tobacco use; in the moderate-risk zone for healthy diet and blood pressure; and the high-risk zone for physical activity and exercise. Physicians exhibited higher levels of CVRFs compared to other healthcare professionals. Furthermore, high socioeconomic status was associated with a high risk of CVD. Our findings identify opportunities for targeted training and effective interventions to reduce the burden of CVDs among HCPs and beyond and maximize their potential as health educators and influencers with their patients and students.

## Introduction

Cardiovascular diseases (CVDs) refer to a group of disorders of the heart and blood vessels and they include coronary heart disease, cerebrovascular disease (stroke), and rheumatic heart disease [1]. CVDs constitute a major public health concern worldwide, accounting for a significant proportion of morbidity and mortality [2-4]. Globally, CVD-related mortality increased from 12.4 million in 1990 to 19.8 million in 2022 and is projected to further increase to 23.6 million by 2030 [1]. In the past decades, about 80% of CVD deaths worldwide occurred in developing countries [5]. In 2019, more than one million deaths were attributable to CVD in sub-Saharan Africa, which constituted 5.4% of all global CVD-related deaths and 13% of all deaths in Africa [5]. Like many African countries, Cameroon is facing the scourge of CVD, with the condition linked to 12% of total deaths in 2012 [6].

Cameroon has witnessed a significant rise in CVDs, which has been associated with epidemiological transition and the adoption of Westernized lifestyles [4]. This surge is closely linked to increasing CVD risk factors (CVRFs), with some statistics documented to be above continental and global levels, such as for hypertension, diabetes, and obesity [7-10]. Tobacco use among males aged 15 years and above is 43.8%, surpassing the global average of 36.1%; and 28.5% of adults in Cameroon are physically inactive, slightly above the global rate of 27.5% [11]. In 2017, 37% of Cameroonian women over 25 were classified as overweight or obese [11]. Despite this alarming CVD burden, a community-based study has highlighted poor awareness of CVD and its risk factors among the population [12]. Improved awareness is essential for effective prevention and management of CVD in Cameroon.

Many cases of CVDs can be prevented by addressing known CVRFs such as smoking, unhealthy diet, obesity, alcohol consumption, and physical inactivity [2,13]. Healthcare professionals (HCPs) are an essential group of professionals whose work is critical to improving and sustaining maximal health within society [14]. Owing to their specialized training, HCPs are expected to be role models and demonstrate a higher degree of awareness in terms of improving and maintaining good health through their lifestyles, including the reduction of CVRFs [15-16]. However, there is increasing evidence that the burden of CVRFs among HCPs is high, with Hedge et al. in India reporting a 31.2% prevalence of CVRFs in this cohort [17]. A higher prevalence (50%) has been reported in Ghana with several HCPs having at least one known cardiometabolic risk [16]. 

The higher prevalence of CVRFs among HCPs has been associated with greater risks of musculoskeletal conditions, higher incidence of CVDs, and increased morbidity and mortality impacting workforce capacity [14-15]. There is growing evidence to suggest that HCPs' health behaviors and CVRFs can influence the advice they provide to patients [14,18]. However, there is scarce data on the CVRF profile of HCPs within hospital settings in Cameroon [4,14]. Hence, this study aimed to evaluate the pattern of lifestyle-related CVRFs among HCPs at the Bamenda Regional Hospital (BRH), in Bamenda, Cameroon, by using the established Health Improvement Card (HIC). This study aimed to address the following research questions: (1) what is the prevalence of various CVRFs among HCPs at the BRH, and (2) What is the pattern of these risk factors among the different HCPs at the BRH?

## Materials and methods

Study design

We conducted a hospital-based cross-sectional survey to assess lifestyle-related CVRFs among HCPs at BRH, Bamenda, Cameroon from July 1 to September 30, 2024.

Setting

BRH is a third-level referral hospital in the Northwest Region of Cameroon, located in the Bamenda II subdivision of Mezam Division. Currently, it has over 500 medical personnel, supplemented by a high volume of biomedical students constantly on internships or placements. BRH has four main departments (internal medicine, surgery, pediatrics and obstetrics, and gynecology), several specialized units (emergency, intensive care, physiotherapy, ophthalmology, dental, tuberculosis, HIV), and an integrated women’s center. Each department and service employ a range of HCPs including doctors, nurses, physiotherapists, and laboratory technicians.

Study population

The study involved HCPs working at BRH during the study period. All adult HCPs of both genders aged above 21 years working across all services and present at the time of data collection were eligible. Out of the total 602 staff at the BRH, only those who consented to participate in the study were included.

Sampling and sample size 

A non-probability (convenience) sampling method was used for this survey. Although a random sampling method could have offered more rigor and minimized selection bias, a convenience sampling method was deemed more feasible and practical given the limited research resources available at the time. A minimum sample size of 205 participants was estimated based on the prevalence (19.5%) of CVRFs among patients in our setting [4]. A minimum sample size for statistical significance was calculated using Cochran’s formula based on a study conducted in the Southwest Region of Cameroon [4].

Survey instrument

The modified HIC was used for data collection in this survey [19]. This evidence-informed HIC tool was developed by the World Health Professions Alliance, an alliance of the five leading health professions in the world. It has since been modified to reflect the literature [19]. The HIC consists of two pages with demographic and biometric data on one page, and lifestyle practices on the other. It uses a traffic light color-coded system to identify low, medium, and high health risks. It is a simple assessment and educational tool to address common CVRFs [20-21]. The sociodemographic data we collected included age, sex, profession/duties, marital status, religion, residence, and socioeconomic status. The lifestyle data included dietary habits, physical activity, and tobacco and alcohol use.

Not all variables on HIC were captured as the tool was used mainly for the evaluation of the CVRFs of the participants. The original HIC consists of several sections including patient biometric data for fasting blood sugar and cholesterol level; and written commitment by the patient and the health professional to effect change in unhealthy biometric parameters or lifestyle behaviors which were omitted in the modified HIC for the current study. These aspects were omitted because of the invasive nature and the associated cost of blood tests with minimal influence on the study objectives. The HIC scores were calculated by using assigned figures to each parameter based on specific criteria, which cumulatively reflect an individual’s overall health status. For instance, with BMI as a parameter, scores were based on weight categories (normal, overweight, obese), with normal as 1, overweight as 2, and obese as 3. The higher the HIC score, the higher the health risk. The level of health and/or disease risk was symbol-coded as high-risk (†), caution/moderate-risk (#), and healthy/low-risk (*) zones [19-21].

Data collection

Data were collected from July 1 to September 30, 2024. The study's aim and purpose, along with the potential benefits of participation, were explained to each potential participant. They were given the opportunity to ask any questions they had. Informed consent was obtained from all participants included in the study. Data collection was based on data extraction from the modified HIC on diet, physical activities, smoking, and alcohol consumption. For diet and fruit portion sizes, we used the World Health Organization definition of a portion (fruits or vegetables as approximately 80 grams) with individuals consuming at least five portions (400 grams) per day [20,22]. Practically, we liken one portion of fruits to approximately one medium-sized fruit like apple, banana, orange, or a handful of berries or grapes. For vegetables, a small bowl of salad or half a cup of cooked vegetables [20,22]. 

We recorded blood pressure based on established methods including a 15-minute rest period before obtaining the first blood pressure measurement. Measurement was done thrice in sitting based on current recommendations of the International Society of Hypertension guidelines [23]. All blood pressure measurements were conducted by a trained clinician (ABW) using a mercury sphygmomanometer with a properly sized cuff (A&D Medical UM-102A Mercury-Free Sphygmomanometer). The weight and height of all participants were collected using a standard weight (mechanical weighing scale) and stadiometer respectively and used to calculate BMI. Data were also collected on socioeconomic status and participants were classified as lower class if they earned below 170000 CFA Francs (267 USD), middle class if they earned more than the lower class but below 460,000 CFA Francs (723USD), and upper class if they earned above 460,000 CFA Francs per month. These ranges were based on the Modified Kuppuswamy Scale for the year 2022 [24].

Data management and analysis

Throughout the study period, only research team members had access to the raw data stored and retained on a secure, password-protected computer. The data were then transferred to SPSS Statistics version 26 (IBM Corp., Armonk, NY) for statistical analysis [25]. Descriptive statistics (frequencies, central tendency, dispersion/variation, and percentages) were used to present the demographic information and pattern of CVRFs among participants. Independent t-test and Chi-square analysis were used to compare continuous and categorical variables respectively with statistical significance set at a p-value <0.05. All missing data were excluded from the analysis.

Ethical and administrative considerations

This study was conducted following the Declaration of Helsinki [26] and received ethical approvals from the IRB of St. Louis University (SLUI/PHY/IRB/2024/089) and RHB (N0/261/APP/RDPH/RHB/IRB). Relevant authorizations were obtained from the North West Delegation of Health and Regional Hospital Bamenda, and all participants consented to their involvement in the study.

## Results

Participants’ characteristics

A total of 237 participants completed the study and were included in the final analysis (Table 1). Most participants were females (59.1%, n=140). The mean age of the participants was 30.1 ± 5.8 years (range: 20-60 years). Most participants were in the age group of 20-30 years (65%, n=154), with the majority being nurses (56.5%, n=134) in this age group. Nurses constituted the highest proportion of the HCPs (91.6%, n=217). The majority of the participants resided in urban settings and belonged to a middle socioeconomic class (79.7%, n=189).

**Table 1:** Characteristics of the study population (n=237)

Variables	Number (N)	Percentage (%)
Age group, years		
20-30	154	65
31-40	73	30.8
>40	10	4.2
Sex		
Male	97	40.9
Female	140	59.1
Occupation		
Nurse	134	56.5
Medical student (level 7)	55	23.2
Doctor	35	14.8
Physiotherapist	8	3.4
Dentist	3	1.3
Pharmacist	2	0.8
Residence		
Urban	217	91.6
Rural	20	8.4
Religion		
Christian	205	86.5
Muslim	32	13.5
Socioeconomic status		
Lower class	35	14.8
Middle class	189	79.7
Upper class	13	5.5

Lifestyle-related CVD risk factors among healthcare providers 

Four lifestyle factors were assessed based on the HIC color coding and are reported in Table 2. The majority of HCPs reported eating less than five portions of fruits and vegetables per day (54%, n=128): in the moderate risk zone (#); 97.9% (n=232) of the participants reported they had never smoked or were smoking: in the low-risk zone (*). More than two-thirds of the participants reported that physical activity was not part of their daily routine (68.4%, n=162), placing them in the high-risk zone for physical activity.

**Table 2:** Lifestyle and behavioural factors among healthcare workers (n=237) ^*^Goal, or low risk. ^#^Caution, medium risk. ^†^High risk (the risk of developing a CVD increases from the low- to the high-risk level) CVD: cardiovascular disease

Variables	Category	Number (N)	Percentage (%)
Diet	Five portions of fruits and vegetables per day	30	12.7^*^
	Less than 5 portions of fruits and vegetables per day	128	54.0^#^
	I do not eat fruits and vegetables	79	33.3^†^
Physical activity	At least 30 minutes a day	17	7.2^*^
	Less than 30 minutes a day	58	24.5^#^
	Physical activity is not part of my daily routine	162	68.4^†^
Tobacco use	No, I never use or have stopped using tobacco	232	97.9^*^
	Yes, I use tobacco	5	2.1^†^
Alcohol consumption	Less than 2 drinks per day	195	82.3^*^
	3 to 4 drinks per day	42	17.7^#^
	More than 5 drinks per day or >5 days per week	0	0.0^†^

Biometric risk factors among healthcare providers

Table 3 provides the details of BMI and blood pressure in participants based on the HIC card color coding. Approximately half of the participants (49.8%, n=118) were of normal weight (in the low-risk zone), while 47.3% (n=112) were in the medium-risk zone for blood pressure.

**Table 3:** Biometric evaluation of healthcare workers (n=237) ^*^Goal, or low risk. ^#^Caution, medium risk. ^†^High risk (the risk of developing a CVD increases from the low- to the high-risk level) CVD: cardiovascular disease

Variables	Category	Number (N)	Percentage (%)
Body mass index, kg/m^2^	18.5-24.9	118	49.8^*^
	25-29.9	100	42.2^#^
	30 or greater	19	8.0^†^
Blood pressure, mmHg	Systolic blood pressure less than 120 mmHg and/or diastolic blood pressure less than 80 mmHg	109	46.0^*^
	Systolic blood pressure 120-139 mmHg and/or diastolic blood pressure 80-89 mmHg	112	47.3^#^
	Systolic blood pressure of more than 140 mmHg and/or diastolic blood pressure of more than 90 mmHg	16	6.8^†^

Cardiovascular disease risk profile based on Health Improvement Card scores at the Bamenda Regional Hospital

The HIC scores ranged from 6 to 17, with a mean of 10.2 ± 1.7. The higher the HIC score, the higher the CVD risks.

**Table 4:** Cardiovascular disease risk profile based on Health Improvement Card scores at BRH BRH: Bamenda Regional Hospital; SD: standard deviation

Variable	Groups	Health Improvement Card scores, mean ± SD	P-value	t-value	F-value
Gender	Females (n=140)	10.2 ± 1.7	0.781	0.144	
	Males (n=97)	10.3 ± 1.8			
Age group, years	20-30	9.9 ± 1.6	0.000		10.8
	31-40	10.8 ± 1.7			
	>40	11.5 ± 1.4			
Religion	Christian	10.3 ± 1.7	0.394	1.320	
	Muslim	9.8 ± 1.9			
Occupation	Doctor	11.0 ± 1.8	0000		9.219
	Pharmacist	11.0 ± 1.4			
	Nurse	10.5 ± 1.7			
	Physiotherapist	8.0 ± 1.1			
Socioeconomic status	Upper class	11.5 ± 1.3	0.013		4.209
	Middle class	10.0 ± 1.7			
	Lower class	10.0 ± 1.9			

## Discussion

To our knowledge, this is the first study to evaluate the lifestyle-related CVRFs and risk profile among HCPs at BRH. The findings highlight a young workforce with diet and physical inactivity as primary CVRFs with tobacco and alcohol use constituting little risk. The biometric scores highlight moderate risk among HCPs at BRH. Our cohort had a mean age of 30.1 ± 5.8 years, similar to 32.1 ± 8.9 and 32.4 ± 8.4 years reported in Ghana and Malaysia, respectively [16,27]. Conversely, higher mean ages of 35.1 ± 11.6 and 39.3 ± 7.4 years have been reported from India and Nigeria [17,28]. The differences may be associated with the varying sample sizes and heterogenous nature of HCPs included in these studies. The young working population in the present study underscores the importance of early screening and lifestyle interventions among HCPs.

Consistent with previous research from Ghana, Nigeria, India, and Malaysia, most participants in our study were female [16-17,27-28]. This may be associated with the fact that most participants were nurses and nursing remains a female-dominated profession [28]. This has wide implications for monitoring and instituting lifestyle interventions given the double roles most nurses hold within their profession and family. In line with prior research, we found a higher proportion of participants (54.0%) in the moderate-risk zone (#) for diet, similar to that reported in Nigeria (56.7%) and China (73%) [21,28]. Conversely, a higher proportion of participants in the low-risk group were reported from India [17]. The differences may be attributed to the cultural and religious influence on dietary patterns and habits in India. This is further enhanced by the availability of national food-based dietary guidelines in India, emphasizing the benefits of healthy fruits and vegetables [29-30]. The absence of local evidence-based knowledge on diet constitutes a lack of a reference point for dietary education both to HCPs and the general public in Cameroon.

Similar to findings reported from Nigeria on physical activity, many of our participants (68.4%) were in the high-risk zone, reflecting physical inactivity [28]. Conversely, Wu et al. reported that most of their study participants were in the low-risk zone for exercise and physical activity [21]. This may be explained by the lack of physical activity policies and implementation in Cameroon and the lack of safe public spaces and structures to enhance physical activity [31-32]. Also, the lack of public health guidance on physical activity implies a lack of reference for what constitutes optimal and suboptimal levels of activity [33]. Consistent with similar findings in Nigeria, we observed that most participants were in the low-risk zone for tobacco and alcohol use (97.9% and 82.3%, respectively) [28]. This may be associated with the awareness of the danger that tobacco and alcohol use pose to health and well-being. To what extent HCPs understand the risks and can educate others in the clinical setting is a topic for discussion and investigation. 

In line with previous studies from China and Malaysia, most participants were in the low-risk zone for BMI (49.8%) [21,27]. In other African nations, overweight and obesity among HCPs ranged from 31.4 to 44.7%; 23.25 to 27.35% in Nigeria; and 26.5% and 47% (overweight and obesity) in South Africa [16,28,34]. The wide range of overweight and obesity may be associated with Westernization and demographic transition across African countries. Further screening, education, and strategic interventions are warranted.

Furthermore, obesity among the African population in general and HCPs in particular is tilted toward females [16,35]. This could be attributed to the relatively sedentary lifestyle of African women; the cultural association of fatness with beauty, a sign of affluence, and good living; and genetics [28,35]. On the contrary, overweight and obesity are strongly linked to non-communicable diseases such as diabetes, hypertension, CVDs, cancers, and musculoskeletal disorders, increasing overall morbidity and mortality [34-37]. In professional settings, obesity is reportedly linked to weight-related discrimination, frequent absenteeism, presenteeism (health-related work limitations), occupational injuries, short-term disability, and decreased productivity [38-40]. Additionally, obesity is associated with early retirement, which can exacerbate the challenge of retaining healthcare workers, especially in developing countries where their numbers are already limited [41-42].

Regarding blood pressure, we report rates of 6.8% and 47% for hypertension and prehypertension, respectively. This aligns with the reported prevalence of hypertension in Cameroon, ranging from 5.7% in rural through 21.9% in semiurban to 47.5% in urban milieu [7-10]. The high prevalence of prehypertension means significant potential for hypertension in the future. Conversely, higher proportions were reported from Ghana: 16.07% and 52.68% for hypertension and prehypertension, respectively [16]. Both our results and those reported in Ghana are lower than the 17.5-37.5% rate recorded among HCPs in a systematic review of hypertension prevalence in West Africa [43]. The lower prevalence in the current study may also be linked to the overall lower mean age of the sample. Higher prevalence rates have been reported in Nigeria and India [17,28].

The current study highlights the notably high rate of prehypertension among HCPs. Prehypertension presents a crucial opportunity to prevent the condition from progressing to established hypertension. However, if untreated, it could result in an increased cardiovascular burden related to hypertension [44]. We reported a consistent increase of CVRFs with age across age groups, which aligns with previous studies [16-17]. While aging is a well-documented non-modifiable factor for CVD, chronological aging alone is not a strong predictor of CVD provided optimal lifestyle factors are addressed early in life, notably diet, exercise, and stress management [45-46]. It is worth noting that biological aging is an antagonist to cardiovascular health and is mediated by early exposures in life [46]. The impact of biological and chronological aging alone on CVRFs warrants elucidation to distinguish the role of simply just living with adverse lifestyle practices for a long time vs. aging.

The highest level of cardiovascular risk was reported among doctors, with a mean HIC score of 11.0 ± 1.8, and the lowest level was observed among physiotherapists (8.0 ± 1.1, p=0000). Similarly, Hegde et al. have reported higher CVRFs among doctors in India [17]. This may be associated with the relatively sedentary nature of routine activities among doctors compared to the more active role of physiotherapists in Cameroon. The absence of physical and rehabilitation equipment in Cameroonian necessitates that physiotherapists become more manually active. The average age of the practicing doctors is higher compared to physiotherapists and other professionals. However, the heterogeneous distribution of the participants makes it difficult to compare their CVRF profiles comprehensively.

Regarding socioeconomic status, our findings demonstrate a higher CVD risk profile among HCPs in the upper class compared to those in the middle and lower classes. This finding may be attributed to several factors. Increased income may be associated with greater access to processed foods, fueling other CVRFs. Despite the better access to healthcare among individuals with high economic status, CVRFs are largely lifestyle-driven and can be best addressed through interventions targeted at lifestyle practices [47]. In low-resource settings such as Cameroon, increasing income and status may also be associated with increased social demands from family and community members. This may place undue psychological stress on this group. The complexity of these factors and their impact warrant more exploration in further studies.

Strengths and limitations

This is the first comprehensive study from Cameroon to assess CVRFs among HCPs. It offers valuable insights into the CVRF profile of HCPs at BRH, highlighting opportunities for health interventions aimed at improving their health and well-being. The study utilized the original Health Improvement Card, ensuring robust data collection. However, the study has a few limitations. For instance, the convenience sampling method adopted for the study may limit the generalizability of its findings. However, we employed a validated instrument, which is easy to understand, and was administered by a trained clinician (ABW), who clarified the participants's doubts and concerns, thereby minimizing reporting biases. Also, there was a lack of precise categorization of all HCPs, as the sample was not weighted according to the proportion of HCPs in each category.

## Conclusions

Our findings indicate that most HCPs exhibit a moderate CVD risk, with poor dietary habits and physical inactivity as primary contributors, while tobacco and alcohol use remain low. Biometric findings show that they are generally in the low-risk zone based on BMI but in the moderate-risk zone based on elevated blood pressure. Socioeconomically, the upper class had more CVRFs than the middle and lower classes. These findings have the potential to inform both clinical and policy changes, thereby facilitating lifestyle interventions in clinical and community settings. There is a need for more targeted studies related to CVRFs in HCP groups and evidence-based interventions to address the growing burden of CVD in Cameroon. Further research is needed to examine and characterize more context-specific CVRFs in Cameroon.
